# Visuo-tactile heading perception

**DOI:** 10.1152/jn.00231.2022

**Published:** 2022-10-19

**Authors:** Lisa Rosenblum, Alexander Kreß, Jakob C. B. Schwenk, Frank Bremmer

**Affiliations:** ^1^Department of Neurophysics, Philipps-Universität Marburg, Marburg, Germany; ^2^Centre for Mind, Brain and Behavior, Philipps-Universität Marburg and Justus-Liebig-Universität Giessen, Giessen, Germany

**Keywords:** heading, multisensory, self-motion, visuo-tactile

## Abstract

Self-motion through an environment induces various sensory signals, i.e., visual, vestibular, auditory, or tactile. Numerous studies have investigated the role of visual and vestibular stimulation for the perception of self-motion direction (heading). Here, we investigated the rarely considered interaction of visual and tactile stimuli in heading perception. Participants were presented optic flow simulating forward self-motion across a horizontal ground plane (visual), airflow toward the participants’ forehead (tactile), or both. In separate blocks of trials, participants indicated perceived heading from unimodal visual or tactile or bimodal sensory signals. In bimodal trials, presented headings were either spatially congruent or incongruent with a maximum offset between visual and tactile heading of 30°. To investigate the reference frame in which visuo-tactile heading is encoded, we varied head and eye orientation during presentation of the stimuli. Visual and tactile stimuli were designed to achieve comparable precision of heading reports between modalities. Nevertheless, in bimodal trials heading perception was dominated by the visual stimulus. A change of head orientation had no significant effect on perceived heading, whereas, surprisingly, a change in eye orientation affected tactile heading perception. Overall, we conclude that tactile flow is more important to heading perception than previously thought.

**NEW & NOTEWORTHY** We investigated heading perception from visual-only (optic flow), tactile-only (tactile flow), or bimodal self-motion stimuli in different conditions varying in head and eye position. Overall, heading perception was body or world centered and non-Bayes optimal and revealed a centripetal bias. Although being visually dominated, tactile flow revealed a significant influence during bimodal heading perception.

## Graphical abstract

**Figure:**
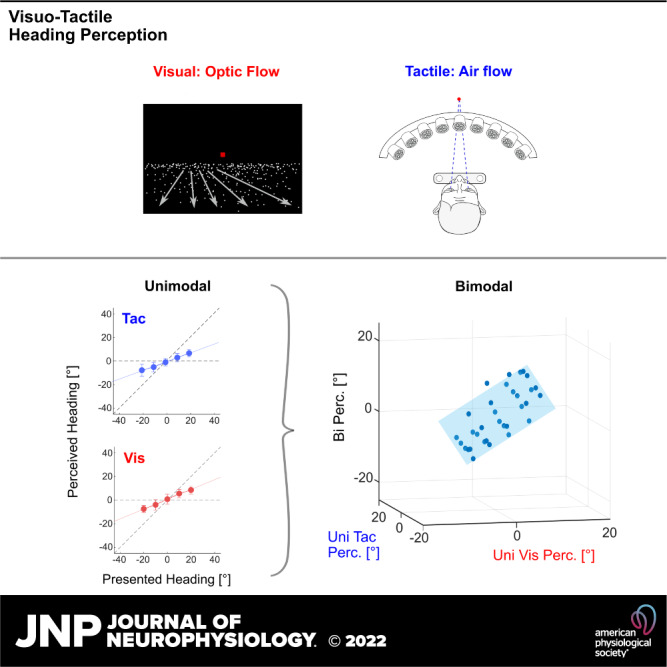

## INTRODUCTION

Successful navigation through an environment requires the accurate estimation of one’s traveled distance (path integration) and direction of self-motion (heading). Previous studies have documented the role of visual (1), vestibular (2), auditory (3), and tactile (4, 5) information for the processing of traveled distance. Studies on heading perception have mainly focused on visual (e.g., Ref. 6) or vestibular (e.g., Ref. 7) stimulation or the interaction of both modalities (e.g., Refs. 8, 9). In the case of directionally congruent visuo-vestibular stimulation, information from both modalities is typically integrated to form a unified perception of heading (10, 11). The role of tactile information in heading perception has only rarely been investigated. Murata and colleagues (12) could show that perception of self-motion can be driven solely by airflow toward the participants’ body. In combination with context-related visual stimuli, airflow has been shown to facilitate vection, i.e., the perception of self-motion (13). In the context of heading, Feng and Lindeman (14) have demonstrated that directional wind can be used as an orientational cue in a spatial orientation task. Finally, in a recent study, we were able to show that tactile heading stimuli (airflow), despite being behaviorally irrelevant, were capable of biasing visually perceived heading. In that study, visual and tactile flow could be either spatially congruent or incongruent (15). Results showed a small but significant attraction toward the tactile flow (distractor), which peaked for an angular separation of 10° between visual and tactile self-motion direction.

In the present study, we tested unimodal (visual or tactile) and bimodal (visuo-tactile) heading perception. In the visual modality, self-motion was simulated as forward motion across a two-dimensional (2-D) ground plane (optic flow). In the tactile modality, self-motion stimuli were delivered by airflow toward the forehead. In separate blocks of trials, participants indicated perceived heading from unimodal visual, unimodal tactile, or bimodal heading information. In bimodal trials, signals from both modalities were presented simultaneously, presenting either congruent heading or headings with an angular separation between visual and tactile heading. Additionally, to estimate the reference frame of visual, tactile, and bimodal heading perception, stimuli were presented in different sessions with varying eye and head orientation.

## METHODS

### Participants

A total of 15 subjects (7 female, 8 male; all right-handed) participated in the experiment. Mean age was 25 ± 5 yr (mean ± SD, range 20–34). All participants had normal or corrected-to-normal vision. The experiment was conducted according to the Declaration of Helsinki and was approved by the local ethics committee. All participants provided written informed consent before the start of the experiment and remained naive to the purpose of the study during the experiment but were offered disclosure thereafter. Experimental sessions took place on six separate days. Testing on each day lasted ∼1.5 h. Participants were compensated for their participation (€8/h).

### Apparatus

Visual stimuli were designed with MATLAB R2019a (The MathWorks, Natick, MA) and the Psychophysics Toolbox (16) running on a Windows PC (Win 10 64-bit; Dell Technologies, Round Rock, TX). Visual stimuli were back-projected on a transparent screen by a video projector (Christie M 4.1 DS + 6 K-M SXGA) at a frame rate of 120 Hz and a resolution of 1,152 × 864 pixels, at 65-cm viewing distance, thereby covering the central 81° by 33° of the visual field. Participants, whose head was stabilized by a chin rest, gave their response via mouse click.

### Visual Stimulus

Self-motion was simulated as forward movement in one of seven directions with constant speed across a virtual 2-D ground plane of 70 white (luminance 100 cd/m^2^) random dots on a black (luminance <0.1 cd/m^2^) background. Dot size and the position of each dot changed dependent on their position relative to the observer with each frame. The maximal lifetime of each dot was limited to prevent participants from orienting on a single dot’s trajectory. After a maximum of 250 ms, a dot disappeared and reappeared at a new random location. We cannot determine distances and speeds based on the stimulus display because the size of the dots or the observers’ height above the ground plane are unknown. Therefore, distances and speed are expressed in arbitrary units (AU) and AU per second. To achieve comparable heading performance (similar precision) in the visual and tactile modalities, dot motion coherency was set to 50%, i.e., half of the presented dots moved in random directions, with all motion trajectories simulating forward motion.

### Tactile Stimulus

Airflow was provided from one of nine commercially available hairdryers (CLATRONIC HT 3393; Clatronic, Kempen, Germany) (with the heating system removed). Dryers were installed above and around the participants’ head on a holder with a half-circle shape with a 6-cm distance between the hairdryers, which corresponds to an angular separation of 10°, and an angle of inclination of 30° toward the participants’ forehead. To make sure that the presented heading as induced by airflow across the participants’ forehead could not have been identified by vision of the dryers’ rotating motors, participants were provided with sight protection (matte black cape) mounted on top of protective goggles covering space above 10° of the upper visual field. Airflow was provided with a speed of ∼8.5 m/s. The hairdryers were operated by an ARDUINO MEGA 2560 Rev3 (Arduino, Somerville, MA) connected to the stimulus computer via a USB port and controlled by the “MATLAB Support Package for Arduino Hardware” (https://www.mathworks.com/matlabcentral/fileexchange/47522-matlab-support-package-for-arduino-hardware). The power supply was delivered by three Adafruit Motor Shields v2.3 (Adafruit, New York, NY). Calibration of the visuo-tactile stimulus system guaranteed presentation onset and offset of visual and tactile stimuli within a maximum of 15 ms. To cancel noise caused by the dryer’s motor rotation (75 dB SPL), participants wore in-ear plugs (EAR PD01002; Axisis GmbH, Düsseldorf, Germany). Additionally, white noise (95 dB SPL) was delivered via noise-canceling over-ear headphones (Bose QuietComfort 35 II; Bose GmbH, Friedrichsdorf, Germany).

### Procedure

**Figure 1.**
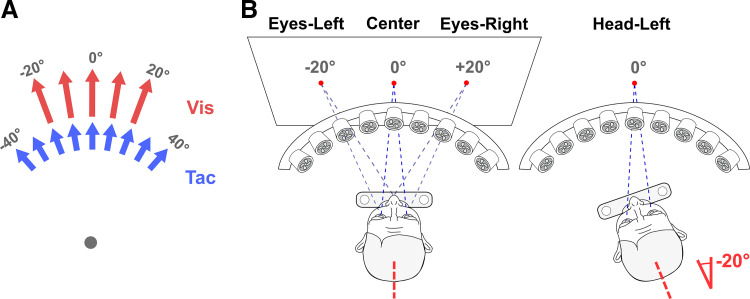
Experimental conditions and setup. *A*: in the visual modality, self-motion stimuli (optic flow) simulated 5 possible heading directions (−20° to 20° in 10° steps). In the tactile modality, self-motion stimuli (airflow) simulated 9 possible headings (−40° to 40° in 10° steps). 0° corresponds to straight-ahead self-motion; negative values indicate motion to the left and positive values motion to the right from straight ahead. Self-motion was presented visual only (Uni Vis), tactile only (Uni Tac), or bimodally. The gray dot indicates the participant’s head position. *B*: in blocks of trials, Uni Vis, Uni Tac, and bimodal heading stimuli were presented in 4 different conditions, in which head and eye orientations were varied. In the Center condition, participants fixated centrally with the head oriented toward straight ahead (0°). In the Head-Left condition, participants fixated centrally with the head on the chin rest rotated 20° toward the left. Head orientation in each condition is shown by the dashed red line. In the Eyes-Left condition, the fixation point was at −20°, i.e., toward the left, with the head centrally aligned. In the Eyes-Right condition, the fixation point was at +20°, i.e., toward the right, with the head centrally aligned. Relative sizes and distances between the tactile stimulation apparatus, participant, and screen are not drawn to scale.

Self-motion was simulated visual only (optic flow), tactile only (tactile flow), or bimodally in separate blocks of trials. In unimodal visual (Uni Vis) trials, stimuli simulated heading directions ranging from −20° to +20° in 10° steps. In unimodal tactile (Uni Tac) trials, directions of airflow ranged from −40° to +40° in 10° steps (Fig. 1). In both cases, negative values indicate headings to the left and positive values heading to the right from straight ahead (0°). Each trial simulated self-motion for 500 ms. Participants conducted 30 trials per heading in both unimodal sets of trials. Figure 1*A* shows all possible headings of both modalities. In bimodal blocks of trials, each visual heading (−20° to +20° in 10° steps) was simultaneously presented with one of seven tactile headings, being either directionally congruent (no offset between visual and tactile heading) or incongruent with an offset of up to ±30°. Visual headings of +20° and −20° were combined with only six tactile headings because of the limited range of tactile flow directions (±40°). This resulted in a total of 5 × 7 − 2 = 33 visuo-tactile stimulus combinations. Each combination was presented 20 times, resulting in a total of 660 trials. After stimulus presentation, participants had to indicate “perceived heading” by placing a mouse-controlled green probe at the appropriate position on a horizontal line spanning the full width of the screen. The initial position of this probe was randomized across trials. Participants fixated a red fixation point on the screen during all parts of a trial. Response time was unlimited, and the next trial started automatically after the response was made.

Uni Vis, Uni Tac, and bimodal blocks of trials were presented in four different combinations of head and eye orientation (Fig. 1*B*): Center condition: participants’ head and eyes centrally aligned, oriented toward straight ahead (fixation 0°, head 0°); Head-Left condition: head rotated 20° toward the left while eyes fixated centrally (fixation 0°, head −20°) (appropriate rotation of the chin rest allowed for stabilizing head orientation in this condition); Eyes-Left condition: fixation −20° toward the left from straight ahead (fixation point location −20°) with head orientation straight ahead (fixation −20°, head 0°). These conditions were completed by all participants (*N* = 15). To probe for the spatial symmetry of potential effects, seven participants were also tested in the Eyes-Right condition: fixation +20° toward the right from straight ahead (fixation point location +20°) with head orientation straight ahead (fixation +20°, head 0°). In all conditions, participants’ torso was aligned with the center of the setup and oriented toward straight ahead. The combination of Uni Vis, Uni Tac, and bimodal trials with the three head/eye settings tested in all participants (Center, Head-Left, Eyes-Left) resulted in nine experimental conditions, tested on six separate days. Each bimodal block of trials was tested on a separate day, always followed by corresponding unimodal blocks of trials on the next day. For seven participants, additional testing of the Eyes-Right condition resulted in two additional sessions on two separate days. Corresponding unimodal visual and unimodal tactile blocks of trials of one condition were always tested on the same day. The order (Uni Vis–Uni Tac. vs. Uni Tac–Uni Vis) was balanced across participants. In a given block of trials, heading directions were presented in pseudorandomized order. The order of the conditions (Center, Head-Left, Eyes-Left, Eyes-Right) was balanced across participants.

Because of its size and position in front of the participant, the tactile stimulation apparatus prohibited the use of an eye tracker during the experiment. However, we verified visually that fixation behavior was comparable between central and shifted conditions (Head-Left, Eyes-Left, Eyes-Right) in a subset of participants without the tactile stimulation apparatus in place.

### Data Processing

For all analyses, a *P* value of 0.05 or smaller indicated statistical significance. For repeated-measurements analysis of variance (ANOVA), Greenhouse–Geisser correction was applied to *P* values in the case of violated sphericity assumption (Mauchly test *P* < 0.05). Effect sizes are reported by η^2^.

#### Multisensory integration.

For each participant, we calculated for the bimodal task and directionally congruent visual and tactile self-motion stimuli the standard deviations of perceived headings. In the case of optimal integration of heading stimuli in a Bayesian sense, responses for bimodal stimuli should show smaller standard deviations (i.e., a higher precision) compared to the smallest standard deviations (highest precisions) of the responses to both unimodal stimuli (17). Predicted bimodal standard deviations ($\sigma_{\mathrm{biOpt}}^{2}$) were calculated from the observed unimodal standard deviations (σ_vis_ for visual and σ_tac_ for tactile stimuli):

(*1*) $$\sigma_{\mathrm{biOpt}}=\;\sqrt{\frac{\left(\sigma_{\mathrm{vis}}^{2}\;\times \;\sigma_{\mathrm{tac}}^{2}\right)}{\left(\sigma_{\mathrm{vis}}^{2}\;+\;\sigma_{\mathrm{tac}}^{2}\right)}}\;$$

Note that this estimation does not take into account variability in the data that stems from nonsensory sources, such as motor noise, in the subjects’ response. However, any such noise is likely negligible in comparison to the sensory uncertainties in this type of task [as shown previously for a similar continuous report in vestibular heading estimation (8)].

#### Impact of single-modality stimuli on bimodal heading perception.

To investigate the impact of visual and tactile stimuli on bimodal heading perception, perceived headings of bimodal trials were entered into the following multiple regression model:

(*2*) $$z\;=\;m_{\mathrm{vis}}\cdot x\;+\;m_{\mathrm{tac}}\cdot y\;+\;m_{\text{vi}{\text{s}}^{2}}\cdot x^{2}+m_{\mathrm{ta}{\text{c}}^{2}}\cdot y^{2}+m_{\mathrm{int}}\cdot x\cdot y\;+\;b$$

Here, for each participant, perceived heading in bimodal trials (*z*) was modeled as a function of perceived heading during pure visual (*x*) and tactile (*y*) stimulation, comprising linear (*x*, *y*), interaction (*x* × *y*), and quadratic (*x*^2^, *y*^2^) terms, and an intercept (*b*). To identify the optimal model for explaining perceived headings of each condition, parameters of the multiple regression model were removed successively, evaluating at each step whether the further model reduction was justified with the Bayesian information criterion (BIC) (18). The BIC depends on the model residuals (RSS) and the number of free parameters (*k*) entered in the model, with smaller BIC values indicating higher model fits:

(*3*) $$\text{BIC}=n\text{ ln}\left(\frac{\mathrm{RSS}}{n}\right)+k\text{ ln}(n)$$

with *k* = number of parameters estimated by the model, *n* = number of data points (sample size), and RSS = residual sum of squares of the model.

## RESULTS

### Unimodal Heading Perception

**Figure 2.**
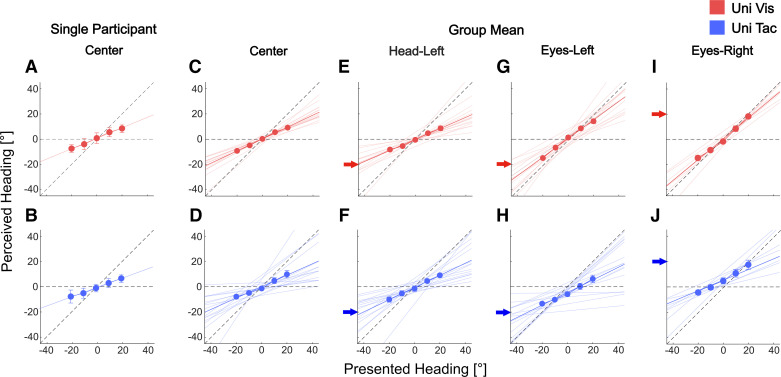
Data and fitted linear regression models for perceived heading in unimodal trials. *Top* (red): data for the visual-only (Uni Vis) condition. *Bottom* (blue): data for the tactile-only (Uni Tac) condition. *A* and *B*: perceived heading (ordinate) as a function of presented heading (abscissa) of 1 participant averaged over trials in the Center condition. Error bars represent SD over trials. Solid lines show linear regressions fitted to the perceived heading. *C* and *D*: mean perceived heading averaged over participants for the Center condition. Light red and blue lines represent linear regression fits to data of single participants. *E* and *F*: mean perceived heading averaged over participants for the Head-Left condition. *G* and *H*: mean perceived heading averaged over participants for the Eyes-Left condition. *I* and *J*: mean perceived heading averaged over participants for the Eyes-Right condition. Error bars represent SE over participants but in most cases were smaller than the symbol size. In all cases, arrows on the ordinate mark either head or eye position in that condition.

In unimodal trials, participants reported perceived heading for visual-only (optic flow) or tactile-only (airflow) stimuli in four conditions, varying in head/eye orientation (Center, Head-Left, Eyes-Left, Eyes-Right). Figure 2 shows representative data from a single participant for the Center condition (Fig. 2, *A* and *B*) and group data averaged across participants for all four conditions (Fig. 2, *C–J*). Mean perceived heading (ordinate) is shown as a function of presented heading (abscissa) for unimodal visual (Fig. 2, *top*; data shown in red) and unimodal tactile trials (Fig. 2, *bottom*; data shown in blue). Here, for the tactile modality, for comparison reasons we only considered headings that had also been presented in the visual modality (−20° to +20° in steps of 10°). The diagonal dashed lines in Fig. 2 indicate veridical heading performance. We fitted data with linear regression functions: *y* = *mx* + *b*, with *y* = perceived heading, *x* = presented heading, *m* = slope, and *b* = intercept. Concerning the slope *m*, a value around *m* = 1.0 would indicate close to veridical performance (assuming an intercept *b* close to 0.0), whereas values of *m* < 1.0 or *m* > 1.0 would indicate a bias of perceived heading toward (centripetal bias) or away from (centrifugal bias) straight ahead, respectively.

#### Single-participant data.

For the single participant (Fig. 2, *A* and *B*), performances in the visual and tactile conditions were rather similar, as indicated by similar slopes in the Uni Vis (*m*_vis_ = 0.41) and Uni Tac (*m*_tac_ = 0.37) conditions. This similarity was intended to allow for testing for a Bayesian integration-based prediction of the performance in the bimodal condition (see methods for details). In both modalities, intercept values were close to zero (*b*_vis_ = 0.06 and *b*_tac_ = −0.08; both linear regressions *P* < 0.001).

#### Group data.

The colored symbols in Fig. 2, *C–J*, show the mean (±SE) across participants of perceived heading for both modalities and all four conditions. The solid thick colored lines show the linear regression fit to these average values. The thin colored lines depict the fit to the single-participant data. In the Head-Left, Eyes-Left, and Eyes-Right conditions, colored arrows on the *y*-axis indicate the respective head or eye position. Linear regressions were statistically significant (*P* < 0.001) in all conditions. Slopes of the linear regression fits as derived from Uni Vis (Fig. 2, *C*, *E*, *G*, and *I*) and Uni Tac (Fig. 2, *D*, *F*, *H*, *J*) data did not differ significantly between the modalities [*m*_vis_Center_ = 0.49, *m*_tac_Center_ = 0.49, *m*_vis_Head-Left_ = 0.43, *m*_tac_Head-Left_ = 0.53, *m*_vis_Eyes-Left_ = 0.7, *m*_tac_Eyes-Left_ = 0.4, *m*_vis_Eyes-Right_ = 0.83, *m*_tac_Eyes-Right_ = 0.54; all *t* < 2.6 and all *P* > 0.0125 (= 0.05/4) with Bonferroni correction for multiple comparisons]. To probe the spatial symmetry of a potential effect of an eye orientation away from straight ahead, a subsample of participants also completed the Eyes-Right condition (*N* = 7). Slopes in the Eyes-Left and Eyes-Right conditions were not significantly different for either the Uni Vis [*m*_Eyes-Left_ = 0.7, *m*_Eyes-Right_ = 0.83; paired *t* test, *t*(6) = −1.26, *P* = 0.255] or the Uni Tac (*m*_Eyes-Left_ = 0.4, *m*_Eyes-Right_ = 0.54; paired *t* test, *t*(6) = −0.95, *P* = 0.38] trials. In Uni Vis trials, slopes of all four conditions were comparable, with one exception: Average heading performance in the Eyes-Right condition showed the steepest slope of the regression [*F*(3,18) = 15.691, *P* < 0.001, η^2^ = 0.723]. In Uni Tac trials, slopes were comparable, i.e., not significantly different between all four conditions [*F*_GG_(1.248,7.489) = 0.552, *P* = 0.518, $\eta_{\mathrm{GG}}^{2}$ = 0.084].

Intercept values (*b*) in all four conditions (Center, Head-Left, Eyes-Left, and Eyes-Right) and both modalities (visual and tactile) did not differ significantly from zero (all *P* > 0.627, mean intercept values over all modalities/conditions: 0.08), with one exception: Mean intercept values of −3.8° (*P* < 0.01) in Uni Tac trials in the Eyes-Left condition (Fig. 2*H*) and 5.3° (*P* < 0.01) in the Eyes-Right condition (Fig. 2*J*) indicated that average perceived heading in this conditions was shifted toward the eye orientation.

### Bimodal Heading Perception

**Figure 3.**
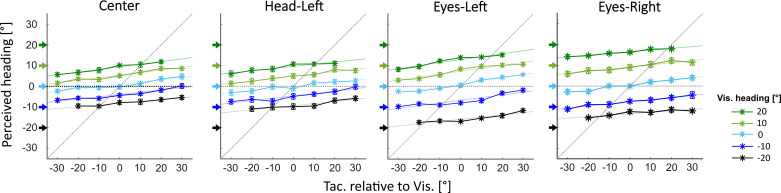
Heading estimation in bimodal trials. In bimodal trials, visual (Vis) and tactile (Tac) heading stimuli either simulated congruent headings or had a maximum offset of 30° between them. Mean perceived headings (ordinate) are shown for each visual heading separately (colored lines) as a function of tactile headings relative to visual heading (abscissa). Colored arrows indicate visually presented headings. Error bars represent SEs over participants.

In bimodal trials, a tactile (airflow) and a visual (optic flow) self-motion stimulus were presented simultaneously, either directionally congruent or incongruent with a maximum offset of 30°. Figure 3 shows mean perceived heading as function of the directional offset between visual and tactile heading stimuli for the four experimental conditions. In each panel, differently colored symbols and lines depict the results for a given visual heading. Colored arrows on the ordinate indicate visually presented headings. If perceived heading in the bimodal condition was independent from the tactile stimulus, all colored lines are flat (slope = 0.0). Likewise, diagonal lines with slope = 1.0 would indicate a strict correlation between tactile information and perceived bimodal heading.

Slopes of *m* > 0 of single regression lines (representing each visually presented heading) indicate that stimuli from both modalities were used for heading estimation in all conditions (all *P* < 0.001). Slopes of single linear regression did not differ between all four conditions [*F*(3,18) = 0.43, *P* = 0.734, η^2^ = 0.067], and intercept values (average spacing between regression lines) were comparable between all four conditions [*F*(3,18) = 1.863, *P* = 0.172, η^2^ = 0.237]. Perceived headings in the Center condition (mean slope: 0.11, average spacing between regression lines: 3.6°) and in the Head-Left trials showed a similar overall pattern: mean slope: 0.11, average spacing between regression lines: 3.9°. Given that the different visual heading stimuli varied by 10° each, an average spacing of only 3.6° between regression lines (together with a mean intercept of ∼0°) is indicative of a centripetal bias. Quantitatively, in line with the data from Uni Vis trials, heading performance showed highest accuracy (i.e., a reduced centripetal bias) in the Eyes-Left condition, as indicated by slightly larger slopes (mean slope 0.13) and wider spacings between regression lines (average spacing between regression lines: 6.3°). In a subsample of participants, an Eyes-Right condition was also measured (mean slope 0.1). Again, spacings between regression lines (average spacing between regression lines: 7.4°) indicated higher accuracy compared with the Center and Head-Left conditions.

To probe for multisensory integration, for each participant we calculated the standard deviations of perceived headings for congruent headings in Uni Vis, Uni Tac, and bimodal trials (−20° to 20° in 10° steps). Next, we derived from unimodal standard deviations corresponding values that would have been expected for bimodal trials (S_biOpt_) if stimulus integration was Bayes optimal. Here, lower standard deviations (i.e., higher precisions) in bimodal compared to unimodal trials would indicate optimal stimulus integration. Average heading performance in none of the four conditions showed such optimal stimulus integration: Observed mean standard deviations in the Center condition (S_bi_ = 6.7°), the Head-Left condition (S_bi_ = 8.4°) and the Eyes-Left condition (S_bi_ = 5.5°) were significantly larger (i.e., lower precision) compared to predicted standard deviations (Center: S_biOpt_ = 4.2°; Head-Left: S_biOpt_ = 4.6°; Eyes-Left: S_biOpt_ = 3.5°) [*F*(1,14) = 46.66, *P* < 0.001, η^2^ = 0.77]. The same applies to heading performance in the Eyes-Right condition that was completed by a subsample of participants. Also here, observed mean standard deviation (S_bi_ = 4.4°) was significantly larger compared to the predicted standard deviation (S_biOpt_ = 3.3°) [*F*(1,6) = 18.5, *P* < 0.05, η^2^ = 0.76].

**Figure 4.**
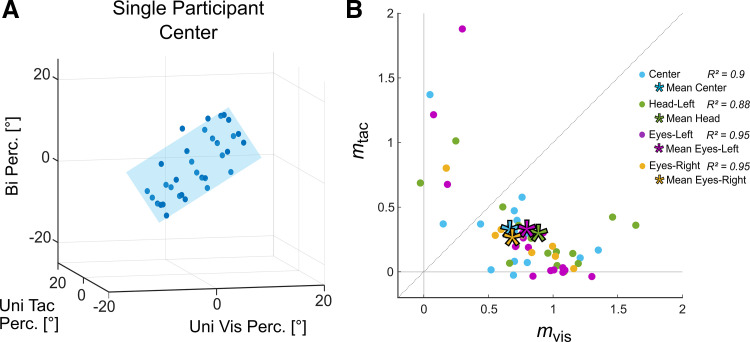
Modeling bimodal heading perception. *A*: perceived headings in bimodal trials (*z*-axis) were modeled with 2-dimensional (2-D) linear regression, with *x* = perceived visual (Vis) heading and *y* = perceived tactile (Tac) heading. *B*: gradients of the 2-D linear regression for the 4 experimental conditions. Data points represent gradients, i.e., slopes (m) in *x* (Vis) and *y* (Tac) directions, of regression planes from individual participants and conditions (Center: blue, Head-Left: green, Eyes-Left: purple, Eyes-Right: yellow). Means over participants are represented by asterisks. Most data points were below the diagonal (*m*_vis_ > *m*_tac_), indicating a stronger weight of visual compared to tactile information.

Although heading stimuli from both modalities were evidently not integrated optimally (in a Bayesian sense), it is apparent from Fig. 4 that sensory stimuli of both modalities were considered by the participants for their heading judgments. To quantify the contribution (or weight) of information from both modalities in bimodal heading perception, perceived headings from unimodal trials (*x*: vis, *y*: tac) were entered as regressors into a multiple regression with bimodal perceived heading as the dependent variable (*z*) for each participant. In all conditions, the full model (as depicted in *Eq. 2*, methods) was successively compared to models without quadratic terms, without the interaction term, and without quadratic and interaction terms (see methods for details). In all conditions, an interaction term, quadratic terms, or both, did not improve model fits compared to the purely linear model. In other words, bimodal heading perceptions in all conditions were best explained by a linear combination of perceived heading in purely visual and tactile trials. Coefficients obtained for this model for each of the conditions are listed in Table 1.

**Table 1.** Mean coefficients derived from the linear regression model

	*N*	*m*_vis_	*m*_tac_	*b*	*R*²
Center	15	0.68	0.33	0.39	0.9
Head-Left	15	0.88	0.3	0.41	0.88
Eyes-Left	15	0.79	0.33	0.8	0.95
Eyes-Right	7	0.72	0.28	0.01	0.95

Perceived headings of bimodal trials (*z*) were predicted by perceived heading from unimodal visual (*x*) and unimodal tactile (*y*) trials, following a 2-dimensional linear regression model (*z* = *m*_vis_ · *x* + *m*_tac_ · *y* + *b*) for each of the 33 bimodal conditions separately. *b*, Intercept; *m*_tac_, mean coefficient for regressor unimodal tactile perceived heading; *m*_vis_, mean coefficient for regressor unimodal visual perceived heading; *R*^2^, mean goodness of fit.

Figure 4*A* shows the 2-D linear regression plane for data of one participant tested in the Center condition. Perceived heading in bimodal trials (*z*-axis) could be predicted (light blue plane) by a linear combination of perceived heading in Uni Vis (*x*-axis) and Uni Tac (*y*-axis) trials (blue dots; *R*^2^ = 0.94).

Figure 4*B* shows the distribution of the gradients, i.e., slopes in *x* (visual) and *y* (tactile) directions, of the 2-D regression planes for all four experimental conditions (Center: blue, Head-Left: green, Eyes-Left: purple, Eyes-Right: yellow). Along a single axis, a value of 1.0 would indicate the same absolute weight as in the unimodal condition. Values smaller than 1.0 would be indicative of a reduced weight. Values larger than 1.0 would indicate a boosted weight of that sensory modality in the bimodal condition. In all conditions, mean values were significantly smaller than 1.0: *m*_Vis,Center_ = 0.684; *m*_Vis,Eyes_Right_ = 0.762; *m*_Tac,Center_ = 0.329; *m*_Tac,Head_Left_ = 0.295; *m*_Tac,Eyes_Left_ = 0.334; *m*_Tac,Eyes_Right_ = 0.272 (all *P* < 0.00625, Bonferroni corrected for multiple comparisons), with the exception of the Head-Left (*m*_Vis,Head-Left_ = 0.8884) and Eyes-Right (*m*_Vis,Eyes-Right_ = 0.7620) conditions (*P* > 0.11) in Uni Vis trials. Most participants showed higher slopes (higher relative weights) for the visual than for the tactile regressor (see Table 1), indicating that the visual modality contributed more strongly to heading judgments in bimodal trials than the tactile modality. Although some participants based their responses completely on visual stimuli and disregarded tactile stimuli (0 on the *y*-axis), most relied on stimuli from both modalities in all conditions. Interestingly, two participants (*subject 4* and *subject 6*) based their responses more strongly on tactile compared to visual stimuli (data points above the diagonal). Goodness of fit (*R^2^*) analyses of fitted linear regression models showed overall high values (*R*^2^ > 0.87) and were comparable between the three conditions that were completed by all participants [Center, Head-Left, and Eyes-Left conditions, *F*_GG_(1.418,19.847) = 3.875, *P* = 0.052, $\eta_{\mathrm{GG}}^{2}$ = 0.21].

### Influence of Head and Eye Positions on Heading Perception

Whereas self-motion through an environment occurs in body-centered coordinates, visual information is sensed in eye-centered coordinates and tactile flow on the head is registered in head-centered coordinates. By varying eye and head orientation for otherwise identical stimulation, we aimed to quantify the influence of the position of the sensors (eye, head) on heading perception. For unisensory conditions, we already showed (Fig. 3) that only for tactile stimuli did the orientation of the eyes away from straight ahead have a significant influence: given a shift of the eyes of 20°, average perceived tactile heading was shifted by 3.8° (19%) and 5.3° (26.5%) for shifts to the left and right, respectively.

**Figure 5.**
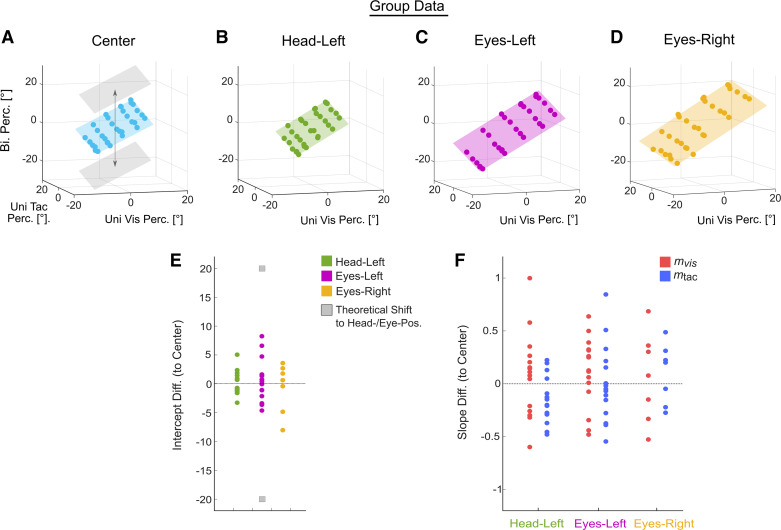
Mean bimodal heading perception as modeled based on unimodal heading perception for all conditions and comparison between fit parameters. Mean perceived (Perc) headings in bimodal (Bi) trials (*z*-axis) were modeled based on perceived heading from unimodal visual (Uni Vis, *x*-axis) and unimodal tactile (Uni Tac, *y*-axis) trials. *A*: transparent gray planes indicate expected shift of perceived heading toward eccentric head or eye position relative to the Center condition (blue plane), if perception was strictly head or eye centered. *B–D*: mean perceived headings in bimodal trials in the Head-Left (*B*), Eyes-Left (*C*), and Eyes-Right (*D*) conditions. *E*: difference of intercept values of each condition and the Center condition for each participant. *F*: difference in slope values [Uni Vis (*m*_vis_): red, Uni Tac (*m*_tac_): blue] between each condition (*x*-axis) and the Center condition. Single points represent data from individual participants.

Figure 5 shows bimodal data. Figure 5, *A*–*D*, depict mean perceived bimodal heading (*z*-axis) as a function of perceived heading in Uni Vis trials (*x*-axis) and Uni Tac trials (*y*-axis) and corresponding linear regression fits. In Fig. 5*A*, the gray planes above and below the Center plane (blue) indicate theoretical shifts in perceived heading for the Head/Eyes-Left and Eyes-Right conditions, if bimodal perception was Head/Eye centered, respectively. In all conditions (Fig. 5, *B–D*), mean perceived heading was not shifted toward head or eye position compared to the Center condition. This is also shown in Fig. 5, *E* and *F*, which show the differences of intercepts (Fig. 5*E*) and slopes (Fig. 5*F*) between the Head/Eyes-Left and Eyes-Right conditions and the Center condition. Difference values for the intercept (mean Head-Left = 0.03, mean Eyes-Left = 0.61, mean Eyes-Right = −0.66) did not differ significantly from zero (1-sample *t* test, all *P* > 0.531), and there was no significant difference between conditions [*F*(1.422,2) = 0.123, *P* = 0.876, η^2^ = 0.02].

Likewise, difference values for the slopes did not differ significantly from zero (1-sample *t* test, all *P* > 0.21) and were comparable between modalities [*F*(1,6) = 0.272, *P* = 0.621, η^2^ = 0.043] and conditions [*F*(2,12) = 0.725, *P* = 0.504, η^2^ = 0.108]. In sum, there was no influence of head and eye position on perceived bimodal heading, pointing toward a body (or world)-centered perception.

## DISCUSSION

In this study we have investigated and compared unimodal visual, unimodal tactile, and bimodal (visuo-tactile) heading perception. By varying head and eye orientation we aimed to determine the reference frame of heading perception.

### Unimodal Heading Perception

In the Center condition, heading stimuli were designed to produce comparable performance accuracies between Uni Vis and Uni Tac trials. Given the experimental apparatus, this was achieved by lowering the coherence of the moving visual stimuli to ∼50%. Perceived headings were biased toward straight ahead in both modalities (i.e., centripetal bias). The same holds true for heading performance in the Head-Left condition. Interestingly, heading performance for Uni Vis trials in the Eyes-Left and Eyes-Right conditions revealed a reduced centripetal bias, whereas it was comparable between all conditions for Uni Tac trials. The bias of perceived heading toward straight ahead is in line with results from the literature [Refs. 15, 19–22, but see also Ref. 8, which found priors to the side (±90°)] and consistent with Bayesian accounts that have proposed a prior for straight ahead as the most common heading direction (21, 23). Another possible explanation might be that participants learned the range of presented headings (likely during the first trials), leading them to adapt their responses to a limited range on the screen (7, 24). Remarkably, we found a reduced centripetal bias (in screen coordinates) of perceived headings in the Uni Vis trials in the Eyes-Left/Right conditions. Although this, in principle, could have been caused by an overall attraction of perceived heading toward the eye, we found no evidence for an eye-centered visual heading perception (see below). Hence, we speculate that heading perception with eccentric gaze might have been perceptually more demanding, leading eventually to an overall better performance.

### Bimodal Heading Perception

In bimodal trials, visual and tactile self-motion stimuli were presented simultaneously, simulating either congruent or incongruent headings with varying angular separation of a maximum of 30° between them. Visuo-tactile heading stimuli were presented in four conditions varying in head and eye orientation (Center, Head-Left, Eyes-Left, Eyes-Right). Perceived heading in all conditions was biased toward (body/world centered) straight ahead (i.e., centripetal bias). Like for the Uni Vis data, the centripetal bias was reduced in Eyes-Left/Right conditions.

Our analysis of response variance revealed that heading stimuli from the visual and tactile modalities were not integrated optimally in a Bayesian sense (25). One possible explanation might be that stimuli of both modalities were inferred to correspond to different sources. Bayesian models of causal inference predict optimal stimulus integration as a function of spatial proximity between stimuli (26–28). However, in our task, participants could have learned already during the first trials that visual and tactile stimuli could present incongruent headings with a large spatial offset between them.

Participants were instructed to “indicate perceived heading” without being asked to focus on one modality in particular. yet participants relied more on visual information, a result in line with previous work on multisensory integration (29, 30). Nevertheless, tactile stimuli were also clearly considered for heading perception. In sum, Bayes-nonoptimal multisensory integration as found in the present study was also reported in previous work (30–33) and can also be found in the case that tactile stimuli are not task relevant (15).

We also found interindividual differences. Although most participants based their heading estimates predominantly on the visual stimuli in all conditions, two participants relied more strongly on tactile stimuli in all conditions. A similar dominance of tactile over visual stimuli has recently been shown by Harris and colleagues (34).

Overall, our bimodal data suggest a consistent influence of both modalities on the integrated heading report in most subjects. It is important to consider this finding in the context of our controlled stimulus environment, which held cue reliability constant. In natural environments, heading cues from both modalities would likely change dynamically. For instance, tactile airflow may provide an unreliable estimate of heading direction when other sources of flow or turbulence are present (e.g., wind or other moving individuals). Previous studies have demonstrated that the relative weights given to individual sensory modalities in heading perception change with their reliability (Refs. 35, 36, both visual/vestibular). Future studies could explore whether this reweighting occurs dynamically on short timescales when tactile airflow varies in reliability.

In our present study, we have tested perception only. Hence, we can only speculate concerning the underlying neural processing. Numerous imaging studies in human observers and neurophysiological studies in the macaque monkey, i.e., the prime animal model of human vision, have documented responsiveness for visual self-motion stimuli in various brain regions (for reviews, see Refs. 37, 38). Among these, the only area also showing responses to tactile stimuli is the macaque ventral intraparietal area (area VIP; Refs. 39, 40). Importantly, neurons with visual and tactile responses have spatially overlapping visual and tactile receptive fields and reveal directionally congruent self-motion responses (41). A functional equivalent of macaque area VIP (hVIP) has also been shown in humans by mean of fMRI (41). Hence, we speculate that neural processing in area hVIP could underly the observed perceptual effects.

### Influence of Head and Eye Position on Heading Perception

We have found no influence of head position or eye orientation on visual heading perception. This was somewhat surprising given previous work providing some evidence for an eye-centered processing of visual self-motion information (monkeys: Refs. 42, 43. humans: Ref. 44). A possible explanation might be the stimulus design. Whereas Crane (44) employed a two-alternative forced choice (2AFC) task, we asked our participants to indicate perceived self-motion direction. Perhaps even more important, Crane (44) also tested the influence of the proportion of visual stimuli in the display showing coherent motion. A reduction in motion coherence induced a shift from eye centered toward screen (or body or world) centered. Hence, it might have been the coherence (roughly 50%) of the moving dots in our displays that contributed toward a body (or world)-centered perception of heading. Given that we have not varied body position, our data do not allow to us dissociate between a body- and a world-centered heading perception.

Unexpectedly, tactile heading perception was modulated by varying eye position. Heading was shifted by 20–25% of the underlying shift in eye position. This is remarkable given that tactile information is initially encoded in a body-centered frame of reference (tactile receptive fields on the skin), and eye position-independent responses have been shown for macaque area VIP (40). We speculate that for eye position away from straight ahead participants were less sure about the tactile stimulation and focused more on where the eye was directed. More experiments, however, would be necessary to better understand the observed effects.

### Study Limitations

A limitation of our approach is the response format, which forced participants to transfer tactile perception into a visual frame of reference. Future studies could aim for a different, for example verbal response format (as employed in, e.g., Refs. 45, 46). Another limitation, leading to higher accuracies for the Eyes-Left/Right conditions might be the size of the screen. When participants fixated 20° to the left/right from straight ahead, screen boundaries might have provided external landmarks. However, the influence of this was likely small, given that screen size was 81° in total, i.e., extending 40° to both sides from the midline. Finally, stimulus duration was rather short. In the case of tactile flow the stimulus could be considered an “air-puff.” Importantly, however, the visual stimuli also only had a duration of 500 ms. In other words, both stimuli were rather phasic. Furthermore, longer durations could have caused other (unwanted) side effects like habituation, memory load, etc. Hence, a 500-ms duration appeared to us to be the best compromise.

### Conclusions

This study demonstrates that tactile as well as visual stimuli are used for heading estimation in a heading estimation task. For a combination of stimuli from both modalities, simulating either congruent heading or with a maximum offset of 30° between stimuli, participants took both sensory signals into account but relied more strongly on visual stimuli. In bimodal trials, varying head and eye position had no significant effect on perceived heading. Only in unimodal tactile trials was heading perception shifted toward eye position but not head position. Since body position relative to the world has been not manipulated in this study, we cannot dissociate between a body- or world-centered frame of reference. Overall, it can be concluded that tactile flow is more important to heading perception than previously thought.

## GRANTS

This work was supported by Deutsche Forschungsgemeinschaft (CRC/TRR 135/A2, project number 222641018, and IRTG-1901-The Brain in Action) and the HMWK cluster project The Adaptive Mind.

## DISCLOSURES

No conflicts of interest, financial or otherwise, are declared by the authors.

## AUTHOR CONTRIBUTIONS

L.R., A.K., and F.B. conceived and designed research; L.R. and A.K. performed experiments; L.R. and J.C.B.S. analyzed data; L.R., J.C.B.S., and F.B. interpreted results of experiments; L.R. and J.C.B.S. prepared figures; L.R. and J.C.B.S. drafted manuscript; L.R., A.K., and F.B. edited and revised manuscript; F.B. approved final version of manuscript.
